# Draft Genome Sequence of the Fungus *Lecanicillium psalliotae* Strain HWLR35, Isolated from a Wheat Leaf Infected with Leaf Rust (Caused by *Puccinia triticina*)

**DOI:** 10.1128/genomeA.01442-17

**Published:** 2018-01-11

**Authors:** Gregory F. S. Harm, Alexie Papanicolaou, William S. Cuddy, Robert F. Park, Michelle C. Moffitt

**Affiliations:** a School of Science and Health, Western Sydney University, Campbelltown, NSW, Australia; b Hawkesbury Institute for the Environment, Western Sydney University, Richmond, NSW, Australia; c Elizabeth Macarthur Agricultural Institute, NSW Department of Primary Industries, Menangle, NSW, Australia; d Plant Breeding Institute, Faculty of Agriculture and Environment, University of Sydney, Cobbitty, NSW, Australia

## Abstract

*Lecanicillium psalliotae* is an entomopathogenic, mycoparasitical, and nematophagous fungus known to produce antibiotic and antifungal compounds. Here, we report the first 36-Mb draft genome sequence of *L. psalliotae* strain HWLR35. The draft genome contains 197 scaffolds and is predicted to have 11,009 protein-coding genes.

## GENOME ANNOUNCEMENT

*Lecanicillium psalliotae*, previously described as *Verticillium psalliotae* (1), is a fungal parasite of many different hosts. Previous work has discovered that *L. psalliotae* is a mycoparasite of soybean rust (caused by *Phakopsora pachyrhizi*) urediniospores (2), is a nematophagous parasite (3), and is entomopathogenic against *Rhipicephalus annulatus* (4). *L. psalliotae* exhibits antagonistic inhibition of its hosts by a variety of means, including the excretion of natural products. *L. psalliotae* is a known producer of oosporein, a red-pigmented dibenzoquinone with antibiotic (5) and antifungal (6) activities. The *L. psalliotae* strain HWLR35 was isolated from a wheat leaf infected with leaf rust (caused by *Puccinia triticina*) from the Plant Breeding Institute, Cobbitty, NSW, Australia. Rust diseases of cereal crops are a threat to global food security (7) and present an interesting opportunity for the implementation of biocontrol agents, such as a fungal mycoparasite. Preliminary identification of *L. psalliotae* HWLR35 was proposed based on internal transcribed spacer (ITS) analysis but has not been confirmed morphologically.

*L. psalliotae* strain HWLR35 was grown on potato dextrose agar from a single-spore culture, and DNA was extracted with the PowerSoil DNA isolation kit (Mo Bio Laboratories, Inc., USA). Genomic DNA was sequenced with 125-bp paired-end reads on the Illumina HiSeq 2500 platform at the Western Sydney University Sequencing Facility. FastQC (8) was used for quality control after the raw reads had adapters removed, and low-quality reads were trimmed through Trimmomatic (9). Blue (10) was used for error correction. To identify the optimum assembly, the error-corrected read data were separately assembled with ABySS (11), SPAdes (12), and SOAP*denovo*2 (13). Error-corrected read data assembled with SOAP*denovo*2 (*k* = 103) performed best according to the following assembly metrics: scaffolds smaller than 1 kb were discarded, and the total assembly length was 36,139,470 bp in 197 remaining sequences, with the largest scaffold being 4,365,396 bp. The *N*_50_ was 2,330,369 bp, the *L*_50_ was 6, the G+C content was 52.74%, and the genome coverage was 80-fold. Two hundred ninety benchmarking universal single-copy ortholog (BUSCO) genes were used to test the genome assembly completeness by gene content (14) and showed that the assembly had 99.3% completeness with 288 single-copy BUSCOs, 2 being duplicated, 1 being fragmented, and 1 missing. TransposonPSI (15) and RepeatModeler (16) were used to mine the genome for transposons and repeats. The genome was masked with RepeatMasker (17) from the mined elements and the complete fungal RepBase data set (18), with 3.33% of the genome masked. Then, GeneMark-ES (19) and Augustus (20) were used for gene prediction in the masked genome, with 11,009 and 11,575 protein-coding genes predicted, respectively. To investigate biosynthetic gene clusters, antiSMASH 3.0 (21) was used, and the program identified 10 type I polyketide synthase (T1PKS) clusters, 9 nonribosomal peptide synthases (NRPS), 4 T1PKS-NRPS hybrid clusters, 5 terpenes, and 6 “other” clusters, for a total of 34 gene clusters. BLASTn (22) of ITS, 5.8S, and partial 18S and partial 28S rRNA sequences from the draft genome revealed a 100% match to *L. psalliotae* strain KYK00175 (GenBank accession no. AB360364) (23). The novel genome sequence of *L. psalliotae* HWLR35 will contribute to biosynthetic gene cluster discovery and help foster research into the genes relating to mycoparasitical interactions.

### Accession number(s).

This whole-genome shotgun project has been deposited in GenBank under the accession no. PHFE00000000. The version described in this paper is the first version, PHFE01000000.

## References

[1] Zare R, Gams W 2001 A revision of *Verticillium* section Prostrata. IV. The genera *Lecanicillium* and *Simplicillium* gen. nov. Nova Hedwigia 73:1–50.

[2] Saksirirat W, Hoppe H-H 1990 Light- and scanning electron microscopic studies on the development of the mycoparasite *Verticillium* *psalliotae* on uredospores of the soybean rust (*Phakopsora* *pachyrhizi* Syd.). J Phytopathol 128:340–344. doi:10.1111/j.1439-0434.1990.tb04283.x.

[3] Yang J, Huang X, Tian B, Wang M, Niu Q, Zhang K 2005 Isolation and characterization of a serine protease from the nematophagous fungus, *Lecanicillium psalliotae*, displaying nematicidal activity. Biotechnol Lett 27:1123–1128. doi:10.1007/s10529-005-8461-0. 16132863

[4] Pirali-Kheirabadi K, Haddadzadeh H, Razzaghi-Abyaneh M, Bokaie S, Zare R, Ghazavi M, Shams-Ghahfarokhi M 2007 Biological control of *Rhipicephalus* (*Boophilus*) *annulatus* by different strains of *Metarhizium anisopliae*, *Beauveria bassiana* and *Lecanicillium psalliotae* fungi. Parasitol Res 100:1297–1302. doi:10.1007/s00436-006-0410-x. 17186273

[5] Wainwright M, Betts RP, Teale DM 1986 Antibiotic activity of oosporein from *Verticillium* *psalliotae*. Trans Br Mycol Soc 86:168–170. doi:10.1016/S0007-1536(86)80133-4.

[6] Nagaoka T, Nakata K, Kouno K, Ando T 2004 Antifungal activity of oosporein from an antagonistic fungus against *phytophthora infestans*. Z Naturforsch C 59:302–304. doi:10.1515/znc-2004-3-432. 15241945

[7] Chen XM 2005 Epidemiology and control of stripe rust [*Puccinia striiformis* f. sp. *tritici*] on wheat. Can J Plant Pathol 27:314–337.

[8] Andrews S 2010 FastQC: a quality control tool for high throughput sequence data. http://www.bioinformatics.babraham.ac.uk/projects/fastqc.

[9] Bolger AM, Lohse M, Usadel B 2014 Trimmomatic: a flexible trimmer for Illumina sequence data. Bioinformatics 30:2114–2120. doi:10.1093/bioinformatics/btu170. 24695404 PMC4103590

[10] Greenfield P, Duesing K, Papanicolaou A, Bauer DC 2014 Blue: correcting sequencing errors using consensus and context. Bioinformatics 30:2723–2732. doi:10.1093/bioinformatics/btu368. 24919879

[11] Jackman SD, Vandervalk BP, Mohamadi H, Chu J, Yeo S, Hammond SA, Jahesh G, Khan H, Coombe L, Warren RL, Birol I 2017 ABySS 2.0: resource-efficient assembly of large genomes using a Bloom filter. Genome Res 27:768–777. doi:10.1101/gr.214346.116. 28232478 PMC5411771

[12] Bankevich A, Nurk S, Antipov D, Gurevich AA, Dvorkin M, Kulikov AS, Lesin VM, Nikolenko SI, Pham S, Prjibelski AD, Pyshkin AV, Sirotkin AV, Vyahhi N, Tesler G, Alekseyev MA, Pevzner PA 2012 SPAdes: a new genome assembly algorithm and its applications to single-cell sequencing. J Comput Biol 19:455–477. doi:10.1089/cmb.2012.0021. 22506599 PMC3342519

[13] Luo R, Liu B, Xie Y, Li Z, Huang W, Yuan J, He G, Chen Y, Pan Q, Liu Y, Tang J, Wu G, Zhang H, Shi Y, Liu Y, Yu C, Wang B, Lu Y, Han C, Cheung DW, Yiu SM, Peng S, Xiaoqian Z, Liu G, Liao X, Li Y, Yang H, Wang J, Lam TW, Wang J 2012 SOAP*denovo*2: an empirically improved memory-efficient short-read *de novo* assembler. Gigascience 1:18. doi:10.1186/2047-217X-1-18. 23587118 PMC3626529

[14] Simão FA, Waterhouse RM, Ioannidis P, Kriventseva EV, Zdobnov EM 2015 BUSCO: assessing genome assembly and annotation completeness with single-copy orthologs. Bioinformatics 31:3210–3212. doi:10.1093/bioinformatics/btv351. 26059717

[15] Haas BJ 2007 TransposonPSI: an application of PSI-Blast to mine (retro-)transposon ORF homologies. http://transposonpsi.sourceforge.net.

[16] Smit AFA, Hubley R 2010 RepeatModeler open-1.0. http://www.repeatmasker.org/RepeatModeler/.

[17] Smit AF, Hubley R, Green P 1996 RepeatMasker. http://www.repeatmasker.org.

[18] Jurka J 2000 Repbase update: a database and an electronic journal of repetitive elements. Trends Genet 16:418–420. doi:10.1016/S0168-9525(00)02093-X. 10973072

[19] Ter-Hovhannisyan V, Lomsadze A, Chernoff YO, Borodovsky M 2008 Gene prediction in novel fungal genomes using an *ab initio* algorithm with unsupervised training. Genome Res 18:1979–1990. doi:10.1101/gr.081612.108. 18757608 PMC2593577

[20] Stanke M, Waack S 2003 Gene prediction with a hidden Markov model and a new intron submodel. Bioinformatics 19:ii215–ii225. doi:10.1093/bioinformatics/btg1080. 14534192

[21] Weber T, Blin K, Duddela S, Krug D, Kim HU, Bruccoleri R, Lee SY, Fischbach MA, Müller R, Wohlleben W, Breitling R, Takano E, Medema MH 2015 antiSMASH 3.0—a comprehensive resource for the genome mining of biosynthetic gene clusters. Nucleic Acids Res 43:W237–W243. doi:10.1093/nar/gkv437. 25948579 PMC4489286

[22] Johnson M, Zaretskaya I, Raytselis Y, Merezhuk Y, McGinnis S, Madden TL 2008 NCBI BLAST: a better web interface. Nucleic Acids Res 36:W5–W9. doi:10.1093/nar/gkn201. 18440982 PMC2447716

[23] Sukarno N, Kurihara Y, Park J, Inaba S, Ando K, Harayama S, Ilyas M, Mangunwardoyo W, Sjamsuridzal W, Yuniarti E, Saraswati R, Widyastuti Y 2009 *Lecanicillium* and *Verticillium* species from Indonesia and Japan including three new species. Mycoscience 50:369–379. doi:10.1007/S10267-009-0493-1.

